# Impact of Abdominal and Thigh Intermuscular Adipose Tissue on Glucose and Cardiometabolic Risk in Adults With Obesity

**DOI:** 10.1210/clinem/dgaf362

**Published:** 2025-07-12

**Authors:** Alba Camacho-Cardenosa, Antonio Clavero-Jimeno, Alessandro Gatti, Manuel Dote-Montero, Mara Concepción, Víctor Manuel Alfaro-Magallanes, Juan J Martin-Olmedo, Rafael Cabeza, Fernando Idoate, José L Martín-Rodríguez, Patricia V García Pérez, Manuel Muñoz-Torres, Jonatan R Ruiz, Idoia Labayen

**Affiliations:** Instituto de Investigación Biosanitaria ibs.GRANADA, 18012 Granada, Spain; Sport and Health University Research Institute (iMUDS), University of Granada, 18071 Granada, Spain; Department of Physical Education and Sports, Faculty of Sport Sciences, Sport and Health University Research Institute (iMUDS), University of Granada, 18071 Granada, Spain; Laboratory of Adapted Motor Activity, Department of Public Health, Experimental Medicine and Forensic Science, University of Pavia, 27100 Pavia, Italy; National PhD Programme in One Health Approaches to Infectious Diseases and Life Science Research, Department of Public Health, Experimental and Forensic Medicine, University of Pavia, 27100 Pavia, Italy; Sport and Health University Research Institute (iMUDS), University of Granada, 18071 Granada, Spain; Obesity and Diabetes Clinical Research Section, Phoenix Epidemiology and Clinical Research Branch, National Institute of Diabetes and Digestive and Kidney Diseases, National Institutes of Health, Phoenix, AZ 85016, USA; Navarre Institute of Health Research, 31002 Pamplona, Spain; Institute for Sustainability & Food Chain Innovation, Department of Health Sciences, Public University of Navarre, 31008 Pamplona, Spain; Navarre Institute of Health Research, 31002 Pamplona, Spain; Institute for Sustainability & Food Chain Innovation, Department of Health Sciences, Public University of Navarre, 31008 Pamplona, Spain; Sport and Health University Research Institute (iMUDS), University of Granada, 18071 Granada, Spain; Department of Physiology, Faculty of Medicine, University of Granada, 18071 Granada, Spain; Department of Electrical, Electronic and Communications Engineering, Public University of Navarre, 31008 Pamplona, Spain; Radiology Department, Mutua Navarra, 31012 Pamplona, Spain; Instituto de Investigación Biosanitaria ibs.GRANADA, 18012 Granada, Spain; Radiology Department, University Hospital Clinic San Cecilio, 18007 Granada, Spain; Radiology Department, University Hospital Clinic San Cecilio, 18007 Granada, Spain; Instituto de Investigación Biosanitaria ibs.GRANADA, 18012 Granada, Spain; Endocrinology and Nutrition Unit, University Hospital Clinic San Cecilio, 18007 Granada, Spain; Department of Medicine, University of Granada, 18071 Granada, Spain; CIBER on Frailty and Healthy Aging, Instituto de Salud Carlos III, 28029 Madrid, Spain; Instituto de Investigación Biosanitaria ibs.GRANADA, 18012 Granada, Spain; Department of Physical Education and Sports, Faculty of Sport Sciences, Sport and Health University Research Institute (iMUDS), University of Granada, 18071 Granada, Spain; CIBER on Physiopathology of Obesity and Nutrition (CIBEROBN), Instituto de Salud Carlos III, 28029 Madrid, Spain; Navarre Institute of Health Research, 31002 Pamplona, Spain; Institute for Sustainability & Food Chain Innovation, Department of Health Sciences, Public University of Navarre, 31008 Pamplona, Spain; CIBER on Physiopathology of Obesity and Nutrition (CIBEROBN), Instituto de Salud Carlos III, 28029 Madrid, Spain

**Keywords:** adipose tissue, abdominal fat, muscle fat, glucose homeostasis, cardiometabolic risk factors, obesity

## Abstract

**Context:**

Intermuscular adipose tissue (IMAT) at different anatomical locations may exert distinct effects on cardiometabolic risk.

**Objective:**

The present study investigated the relationships of abdominal and mid-thigh IMAT with glucose homeostasis and cardiometabolic risk in adults with overweight or obesity.

**Design:**

Multicenter cross-sectional study.

**Setting:**

Outpatient clinic.

**Participants:**

One hundred eighty-nine adults (50% women; age: 46.8 ± 6.3 years) with overweight or obesity (body mass index: 32.9 ± 3.5 kg/m^2^).

**Main Outcome Measures:**

IMAT content in abdominal and mid-thigh regions was measured by magnetic resonance imaging. Mean glucose levels were monitored over 24 hours during 14 days using continuous glucose monitoring devices. We computed a cardiometabolic risk score including fasting high-density lipoprotein cholesterol, triglycerides, glucose, waist circumference, and systolic and diastolic blood pressure.

**Results:**

No associations were identified between abdominal IMAT and glucose homeostasis or cardiometabolic risk (all *P* > .05). In contrast, a positive association of mid-thigh IMAT with 24-hour (β = 0.226; *P* = .007), diurnal (β = 0.224; *P* = .008), and nocturnal mean glucose levels (β = 0.233; *P* = .006) as well as with cardiometabolic risk score (β = 0.324; *P* < .001) was observed. Participants with greater accumulation of IMAT in the mid-thigh compared to the abdominal region exhibited significantly higher mean glucose levels and cardiometabolic risk (all *P* < .005).

**Conclusion:**

These findings emphasize the importance of distinguishing between adipose tissue depots when evaluating cardiometabolic risk, as specific accumulation patterns—particularly in the mid-thigh region—may significantly influence individual risk profiles.

The excessive accumulation of adipose tissue (AT), an inherent characteristic of obesity, is a major risk factor for developing insulin resistance and other cardiometabolic disorders (1). AT is distributed throughout the body in various depots, each with distinct physiological functions (2) and impact on cardiometabolic health (3). While subcutaneous adipose tissue (SAT) is generally considered metabolically benign, ectopic fat accumulation, particularly in abdominal visceral adipose tissue (VAT), is strongly associated with metabolic dysfunction (4-6).

Intermuscular adipose tissue (IMAT) is a fat depot distributed between and around skeletal muscle groups (7). Excessive IMAT accumulation is positively correlated with other fat depots, such as VAT and hepatic fat, both of which are strongly associated with cardiometabolic risk (8, 9). Additionally, IMAT has been strongly associated with the presence of type 2 diabetes, metabolic syndrome (3), cardiovascular disease complications, and hepatic steatosis (10). Indeed, IMAT has been recognized as a key predictor of mortality risk, even in apparently healthy participants (11).

Skeletal muscle is the primary site for glucose oxidation and storage (12). Given its proximity to muscle fibers, IMAT may impair the local muscle environment by secreting inflammatory cytokines, potentially inducing insulin resistance (13). Indeed, increased whole-body IMAT is strongly associated with elevated fasting glucose (14) and reduced insulin sensitivity (15), underscoring its critical role in metabolic dysregulation.

Emerging evidence suggests that the specific anatomic location of IMAT accumulation may differentially impact cardiometabolic health (9, 16, 17). While most research has focused on lower-body IMAT (8, 17-19), less is known about the association between abdominal IMAT and metabolic health (14, 20-22). A particularly intriguing question is whether a greater accumulation of IMAT in the lower body (“pear-shaped”) confers a nonadverse effect similar to that observed with SAT in the same region. Furthermore, the impact of lower-body IMAT on cardiometabolic health, compared to IMAT in abdominal regions, has yet to be thoroughly investigated. This “IMAT phenotype” hypothesis remains largely unexplored and represents a promising area for future research (2). Therefore, the present study aimed to investigate the relationships of abdominal and mid-thigh IMAT with glucose homeostasis and cardiometabolic risk in adults with overweight or obesity.

## Material and Methods

### Study Design and Participants

The present cross-sectional study used data from a parallel-group, multicenter randomized clinical trial conducted in Granada (southern Spain) and Pamplona (northern Spain) (ClinicalTrials.gov identifier: NCT05310721). The aim of the project was to study the efficacy and feasibility of time-restricted eating on VAT (primary outcome), body composition, and cardiometabolic risk factors in adults with overweight or obesity (23). Further details regarding the study rationale, design, and methodology can be found elsewhere (24). The study was registered and approved by the relevant regulatory authorities and ethics committees at each participating center in Spain, including the Andalusian Health Service, the Provincial Ethics Committee of Granada (ref. 1/22), and the Clinical Research Ethics Committee of Navarra (ref PI_2021/119). Informed consent was obtained from all participants prior to their participation.

A total of 189 participants (96 men and 93 women) were included in the analysis. Participants were adults aged 30 to 60 years with overweight or obesity, defined as a body mass index (BMI) between 25.0 and 40.0 kg/m^2^. Additional inclusion criteria were (1) having abdominal obesity defined as waist circumference (WC) ≥ 95 cm in men and ≥82 cm in women, (2i) body weight stability for 3 months prior to study entry, (3) following a sedentary lifestyle, (4) having a habitual eating window ≥ 12 hours, and (5) at least 1 cardiometabolic risk factor for metabolic syndrome. Exclusion criteria were (1) having a medical history with an adverse cardiovascular event, (2) having a disease in which fasting is contraindicated, (3) taking any medications that could modify glucose metabolism, (4) active abuse of tobacco or alcohol consumption, (5) being enrolled in a fasting or weight loss program, (6) working night shifts or having continuous sleep disruptions, and (7) traveling over different time zones during the study period (24).

### Outcomes

#### Body Weight, Body Composition, and AT Depots

Body weight and height were measured using a stadiometer and scale (Seca model 799, Electronic Column Scale, Hamburg, Germany, in both Granada and Pamplona) without shoes and with light clothing. BMI was calculated by dividing body weight in kilograms by height in meters squared. WC was measured following the ISAK procedures (25). Total fat mass was assessed using a dual-energy X-ray absorptiometry (DXA) scan (QDR Discovery Wi Hologic, Inc., Bedford, MA, USA, in Granada and Horizon Wi Hologic, Inc., Bedford, MA, USA, in Pamplona) performed under standardized conditions following the manufacturers’ guidelines for calibration and quality control.

All participants were assessed for specific subcutaneous and IMAT depots with a 3-T magnetic resonance imaging (MRI) scanner (SIEMENS MAGNETOM Vida-XQ Numaris/X VA20A-04N2). Through Dixon-MR images, AT depots were quantified in the mid-thigh area and in the abdominal region between vertebrae L3 and L5 [psoas (L3) and paraspinal and abdominal wall]. Fat and water series images were processed using a semiautomatic, open-source proprietary library called “TisSeg,” developed within our research group, along with its plugin for the open-source software 3D Slicer (www.slicer.org). The “TisSeg” library enabled automatic segmentation of the thigh for the specified slices. A semiautomatic process was used for abdominal segmentation, as it required the identification of the region of interest for the visceral portion of the abdomen. Experienced researchers manually outlined the abdominal viscera, excluding muscular tissue. They also identified the slices corresponding to vertebrae L3 to L5 for the abdominal scans and the mid-femur slice for the thigh, between the femoral head and lateral condyle. Mid-thigh IMAT was calculated as the sum of IMAT (ie, AT between the mid-thigh muscles) and intramuscular AT (ie, AT within the mid-thigh muscles).

**Figure 1.**
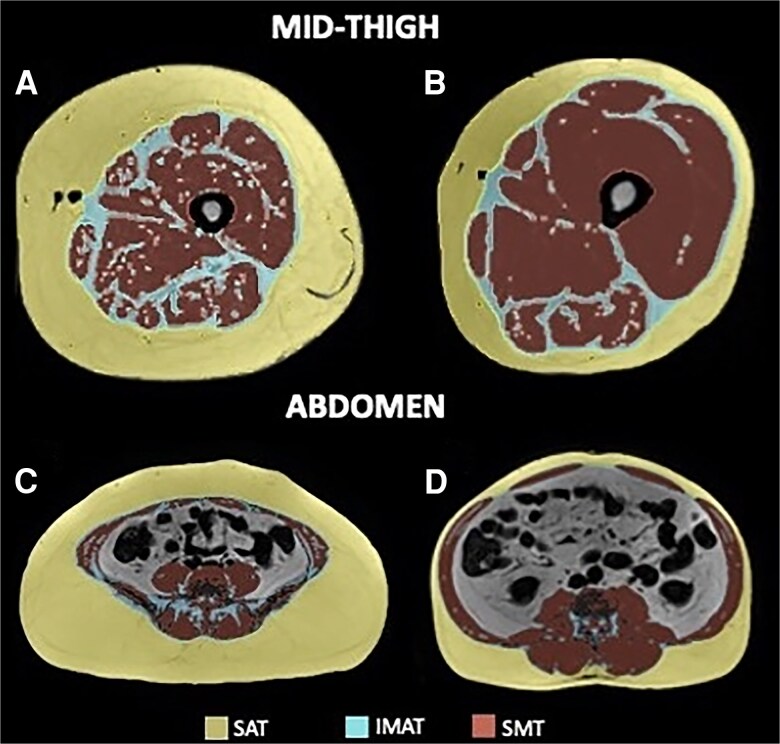
Magnetic resonance imaging. Panels A and B illustrate mid-thigh composition in a woman (49 years of age—A) and a man (45 years of age—B) with a mid-thigh total area of around 350 cm^2^. Panels C and D illustrate abdominal composition in a woman (39 years of age—C) and a man (49 years of age—D) with an abdominal total volume of around 7000 cm^3^. Subcutaneous adipose tissue, IMAT, and skeletal muscle tissue are typically quantified in these axial images, and multiple axial images can be quantified to provide tissue volumes. Abbreviations: IMAT, intermuscular adipose tissue.

As shown in Fig. 1, participants with similar total segment areas (cross-sectional area of the mid-thigh in Fig. 1A and 1B) or volumes (cross-sectional area of the abdomen in Fig. 1C and 1D) have different proportions of each of the deposits. For this reason, abdominal IMAT was standardized by calculating the ratio of IMAT content in the abdominal region to the total abdominal volume, providing a relative measure of abdominal IMAT in relation to the overall abdominal region. Similarly, mid-thigh IMAT was standardized by calculating the ratio of IMAT content in the mid-thigh to the total mid-thigh area. This standardization—expressing abdominal IMAT as a proportion of total abdominal volume and mid-thigh IMAT as a proportion of total mid-thigh area—accounts for individual variability of body size, allowing for more accurate and comparable assessments across participants. Mid-thigh and abdominal SAT were further analyzed [see Supplementary Materials (26)].

#### Cardiometabolic Risk Factors

Fasting blood serum samples were collected and stored at −80 °C to analyze glucose (AU5800 automated analyzer, Beckman Coulter Inc., CA, USA), insulin (UniCel DxI 800 access immune-assay system, Beckman Coulter Inc.), and glycated hemoglobin (HA-8180V® analyzer, A Menarini Diagnostics, Firenze, Italy), as well as high-density lipoprotein cholesterol (HDL-C) and triglycerides (TG) (AU5800 automated analyzer Beckman Coulter Inc.). The homeostasis model assessment of insulin resistance (HOMA-IR) was calculated as described (27).

Systolic blood pressure (SBP) and diastolic blood pressure (DBP) were measured in the morning with an automatic monitor (M3-Comfort, Omron Healthcare Europe B.V. Hoofddorp, The Netherlands, in both Granada and Pamplona), following the 2021 European Society of Hypertension practice guidelines (28).

#### Glycemic Control

Participants' daily glucose levels were measured using continuous glucose monitoring (CGM) devices (FreeStyle LibrePro, Abbott Laboratories, Abbott Park, IL, USA, in Granada and FreeStyle 2; Abbott Laboratories in Pamplona) for 14 days. These CGM models store averages of interstitial glucose approximately every 15 minutes, providing near real-time glucose data through the calculation of total daily mean glucose and values across diurnal and nocturnal windows. Both systems have demonstrated clinically acceptable accuracy, with mean absolute relative difference of ∼12.0% for FreeStyle LibrePro and ∼9.2% for FreeStyle Libre 2. The CGM devices data were time-matched with the triaxial accelerometer (ActiGraph GT3X, Pensacola, FL, USA, in both Granada and Pamplona) data to allow for the calculation of the mean glucose over the diurnal and nocturnal windows. That is, we applied the accelerometer-derived sleep onset and wake-up times to calculate the mean glucose over the full day (namely the 24-hour mean glucose), the waking hours (diurnal mean glucose), and the sleeping hours (nocturnal mean glucose) for each participant's day. The accelerometers were initialized to collect raw accelerations at a frequency of 100 Hz, and participants wore the device on their nondominant wrist. This equipment was configured to measure for 14 consecutive days (24 hours/day). When the 14 days finished, raw data from accelerometers were downloaded using the ActiLife software (ActiGraph LLC), and the raw data were processed with the open-source GGIR R package (version 2.10-3) (22).

#### Cardiometabolic Risk Score

The Cardiometabolic Risk Score (CRS) is a continuous score that includes the metabolic syndrome variables. Sex-specific z-score CRS were calculated using the group SD for each component, with the sum of the z-scores for each component divided by 6 to compile the cardiometabolic risk score with units of SD (29). The equations used to calculate the score were


$$\begin{array}{rl} \mathrm{Me}n^{\prime }s\;\mathrm{CRS} & =[(40\text{–}HDL-C)/SD]+[(TG\text{–}150)/SD]\\ & \;+[(glucose\text{–}100)/SD]+[(WC\text{–}94)/SD]\\ & \;+[(SBP\text{–}130)/SD]+[(DBP\text{–}85)/SD] \end{array}$$

$$\begin{array}{rl} \mathrm{Wome}n^{\prime }s\;\mathrm{CRS} & =[(50\text{–}\mathrm{HDL}-C)/\mathrm{SD}]+[(\mathrm{TG}\text{–}150)/\mathrm{SD}]\\ & \;\;\;+[(\mathrm{glucose}\text{–}100)/\mathrm{SD}]+[(\mathrm{WC}\text{–}80)/\mathrm{SD}]\\ & \;\;\;+[(\mathrm{SBP}\text{–}130)/\mathrm{SD}]+[(\mathrm{DBP}\text{–}85)/\mathrm{SD}] \end{array}$$

A CRS > 0 indicates increased metabolic risk, with higher values reflecting a more pronounced presence of metabolic syndrome components.

#### Confounders

Daily energy intake (kcal · d⁻^1^) was estimated based on 24-hour dietary recalls, using EasyDiet—Programa de Gestión de la Consulta de la Academia Española de Nutrición y Dietética (Biocentury, S.L.U. 2016). At the same time, levels of moderate-to-vigorous physical activity (MVPA) were objectively assessed with ActiGraph GT3X accelerometers (24).

### Statistical Analysis

The sample size for this study was determined based on the primary outcome of the original randomized controlled trial (24). Since the present cross-sectional analysis used baseline data only, no specific sample size or power calculation was performed.

Data were expressed as SD for normally distributed variables or as median (first quartile–third quartile) for nonnormally distributed variables. Nonnormally distributed variables were log-transformed using base-10 logarithms, while normally distributed variables remained untransformed. We conducted a bivariate correlation analysis to examine the relationship between abdominal IMAT/total volume and mid-thigh IMAT/total area with glucose homeostasis and cardiometabolic risk. Next, we computed linear regression analysis to evaluate the relationship between both with glucose homeostasis and cardiometabolic risk after adjusting for age and sex (model 1, except for the CRS, which was adjusted only for age) and further adjusting for age, sex, and total fat mass (model 2, except for the CRS, which was adjusted only for age and total fat mass). As a sensitivity analysis, the analyses were additionally adjusted for total energy intake and MVPA and found that the results remained unchanged. Additionally, those participants with impaired glucose metabolism (fasting glucose ≥ 100 mg/dL) were excluded from the analysis, with similar findings [see Supplementary Tables S1 and S2 (26)]. To further compare the strength of the associations of abdominal and mid-thigh IMAT with cardiometabolic risk and insulin resistance, we calculated a ratio incorporating both depots. This combined approach allowed for a more comprehensive evaluation of the AT distributions and their collective influence on glucose homeostasis and cardiometabolic risk. We categorized participants into 3 groups (tertiles) based on the ratio of the 2 IMAT depots (abdominal/mid-thigh IMAT): low (<33%), medium (33-66%), and high (>66%). Then, we compared the low vs high IMAT ratio groups on glucose homeostasis and cardiometabolic risk using parametric or nonparametric analysis of covariance, as appropriate, adjusting for age, sex, and total fat mass.

To explore the relationships of abdominal IMAT/total volume and mid-thigh IMAT/total area with cardiometabolic risk factors, we employed a structural equation modeling (SEM) for mediation analysis. This SEM allowed us to assess the association of abdominal IMAT/total volume, mid-thigh IMAT/total area with cardiometabolic risk through its components. Specifically, the SEM incorporated direct pathways from abdominal IMAT to HDL-C, TG, fasting glucose, WC, SBP, and DBP. These components were modeled in their z-score forms, as shown in the previous formula above (see Cardiometabolic Risk Score). Indirect pathways were also modeled, where these metabolic markers mediated the effect of abdominal IMAT/total volume and mid-thigh IMAT/total area on the overall cardiometabolic risk score.

For all analyses, a *P*-value <.05 was considered statistically significant. The β coefficient was presented as both nonstandardized and standardized. Analyses were conducted using R software (version 4.4, R Foundation for Statistical Computing). The “ggplot2' package was utilized for linear regression plots, the “lavaan” package for SEM mediation analysis, the “Durga” package for group comparison plots, and the “emmeans” package for post hoc parametric comparisons.

## Results

### Descriptive Characteristics

Table 1 displays the descriptive characteristics of the study participants. Among the 197 participants included initially in the study, a total of 189 were finally included in the present analysis based on available MRI images of the mid-thigh, abdominal region, or both, due to technical errors in image acquisition. Of these, 185 participants had MRI images of the abdominal region, while 180 had MRI images of the mid-thigh region.

**Table 1.** Descriptive characteristics of the study sample

	n (male, female)	All	Men	Women
Age (years)	189 (96, 93)	47.0 (43.3-51.0)	48.0 (44.0-51.3)	46.0 (43.0-50.0)
Weight (kg)	189 (96, 93)	94.9 (85.8-103.0)	100.5 (93.4-110.2)	87.8 (80.1-96.4)
Height (cm)	189 (96, 93)	169.1 (162.5-176.8)	176.4 (172.0-181.6)	162.6 (159.3-166.2)
Body mass index (kg/m^2^)	189 (96, 93)	32.9 (30.7-35.2)	32.4 (30.7-34.8)	33.2 (30.7-36.2)
Abdominal region				
IMAT (cm^3^)	185 (92, 93)	241 (191-319)	275 (214-361)	220 (169-272)
IMAT/total volume	185 (92, 93)	0.037 (0.029-0.048)	0.039 (0.031-0.052)	0.036 (0.025-0.045)
Subcutaneous AT (cm^3^)	185 (92, 93)	2714 (2207-3284)	2555 (2019-3228)	2827 (2408-3394)
Visceral AT (cm^3^)	185 (92, 93)	1242 (926-1655)	1529 (1243-2028)	967 (750-1242)
Mid-thigh region				
IMAT (cm^2^)	180 (93, 87)	26 (21-32)	29 (24-35)	23 (20-28)
IMAT/total area	180 (93, 87)	0.088 (0.076-0.103)	0.098 (0.085-0.112)	0.078 (0.068-0.088)
Subcutaneous AT (cm^2^)	180 (93, 87)	103 (72-148)	73 (57-102)	147 (108-183)
Glucose homeostasis				
Fasting glucose (mg/dL)	189 (96, 93)	93 (88-99)	94 (90-101)	92 (87-97)
Fasting insulin (mU/L)	188 (96, 92)	10.5 (8.1-14.2)	11.3 (8.1-14.7)	10.4 (8.1-13.7)
HOMA-IR	188 (96, 92)	2.5 (1.8-3.4)	2.6 (1.9-3.7)	2.4 (1.8-3.1)
HbA1c (%)	189 (96, 93)	5.3 (5.1-5.6)	5.3 (5.1-5.6)	5.3 (5.1-5.6)
24-hour mean glucose (mg/dL)	178 (89, 89)	105 (100-112)	109 (102-114)	102 (98-108)
Diurnal mean glucose (mg/dL)	178 (89, 89)	106 (102-113)	111 (103-116)	104 (99-110)
Nocturnal mean glucose (mg/dL)	176 (89, 87)	101 (94-109)	105 (98-109)	96 (92-107)
Cardiometabolic risk factors				
HDL-C (mg/dL)	189 (96, 93)	53 (45-61)	50 (43-57)	57 (49-62)
TG (mg/dL)	189 (96, 93)	114 (86-154)	129 (88-167)	104 (84-139)
WC (cm)	189 (96, 93)	101.8 (9.9)	107.5 (7.9)	95.8 (8.1)
SBP (mmHg)	187 (96, 91)	122 (114-134)	126 (118-136)	119 (109-128)
DBP (mmHg)	187 (96, 91)	80 (10)	83 (9)	78 (11)
Cardiometabolic risk score	186 (95, 91)	−0.7 (3.4)	−0.1 (3.2)	−1.4 (3.4)

Data are presented as mean (SD) when normally distributed or median (first quartile–third quartile) when not. The ratio of abdominal IMAT to total abdominal volume was calculated by dividing the abdominal IMAT volume (cm^3^) by the total abdominal cavity volume (cm^3^), thereby accounting for abdominal size. Mid-thigh IMAT is calculated as the sum of IMAT (ie, AT between the mid-thigh muscles) and IMAT (ie, AT within the mid-thigh muscles). The ratio of mid-thigh IMAT to total mid-thigh area was calculated by dividing the IMAT area (cm^2^) by the total mid-thigh area (cm^2^), thereby accounting for mid-thigh size. Cardiometabolic risk score was calculated for men as [(40 − HDL-C)/SD] + [(TG − 150)/SD] + [(fasting glucose − 100)/SD] + [(WC − 94)/SD] + [(SBP − 130)/SD] + [(DBP − 85)/SD] and for women as [(50 − HDL-C)/SD] + [(TG − 150)/SD] + [(fasting glucose − 100)/SD] + [(WC − 80)/SD] + [(SBP − 130)/SD] + [(DBP − 85)/SD].

Abbreviations: AT, adipose tissue; DBP, diastolic blood pressure; HbA1c, glycated hemoglobin; HDL-C, high-density lipoprotein cholesterol; HOMA-IR, homeostasis model assessment of insulin resistance; IMAT, intermuscular adipose tissue; SBP, systolic blood pressure; SMT, skeletal muscle tissue; TG, triglycerides; WC, waist circumference.

### Association of Abdominal and Mid-thigh IMAT With Other AT Depots

Abdominal IMAT/total volume was negatively and significantly associated with abdominal SAT [r = −0.26, *P* < .001, Supplementary Fig. S1 (26)], and mid-thigh SAT (r = −0.35, *P* < .001, Supplementary Fig. S2) but not with VAT [r = 0.07, *P* > .05, Supplementary Fig. S1 (26)]. Mid-thigh IMAT/total area was positively associated with VAT [r = 0.49, *P* < .001, Supplementary Fig. S1 (26)] and negatively associated with mid-thigh SAT[(r = −0.48, *P* < .001, Supplementary Fig. S1 (26)]. As expected, VAT was positively associated with HOMA-IR, 24-hour, diurnal and nocturnal mean glucose levels, as well as with CRS (all *P* < .05). Mid-thigh SAT was negatively associated with 24-hour and diurnal and nocturnal mean glucose levels (all *P* < .05) but not with HOMA-IR or CRS (both *P* > .05).

### Association of Abdominal and Mid-thigh IMAT With Insulin Resistance, Glucose Homeostasis, and Cardiometabolic Risk

Bivariate correlation analysis showed that abdominal IMAT/total volume was not significantly associated with either HOMA-IR; 24-hour, diurnal, and nocturnal mean glucose levels; or CRS [all *P* > .05, Supplementary Fig. S2 (26)], and the results did not change after adjusting for age, sex, and total fat mass (all *P* > .05, model 1, Table 2). In contrast, mid-thigh IMAT/total area was positively and significantly associated with 24-hour, diurnal, and nocturnal mean glucose levels, as well as with CRS [*P* < .001, Supplementary Fig. S2 (26)]. These findings remained after adjustment for age, sex, and fat mass (Table 2). Although the strength of the associations was attenuated, the findings in glucose homeostasis persisted after additional adjustment for VAT [Supplementary Table S3 (26)].

**Table 2.** Associations of abdominal and mid-thigh IMAT with glucose homeostasis and cardiometabolic risk factors in adults with overweight or obesity

		Model 1			Model 2		
		b (95% CI)	β	*P*	b (95% CI)	β	*P*
Abdominal IMAT/total volume	HOMA-IR	−2.220 (−4.637, 0.196)	−0.143	.071	−1.630 (−3.944, 0.683)	−0.105	.166
	24-hour mean glucose (mg/dL)	−0.185 (−0.646, 0.276)	−0.061	.429	−0.133 (−0.594, 0.329)	−0.043	.572
	Diurnal mean glucose (mg/dL)	−0.154 (−0.601, 0.293)	−0.052	.496	−0.106 (−0.553, 0.342)	−0.036	.642
	Nocturnal mean glucose (mg/dL)	−0.234 (−0.806, 0.338)	−0.063	.421	−0.152 (−0.723, 0.419)	−0.041	.599
	Cardiometabolic risk score	−14.544 (−50.567, 21.479)	−0.062	.427	−1.329 (−35.665, 33.006)	−0.006	.939
Mid-thigh IMAT/total area	HOMA-IR	0.313 (−0.033, 0.659)	0.153	.076	0.224 (−0.108, 0.556)	0.110	.184
	24-hour mean glucose (mg/dL)	0.096 (0.030, 0.162)	0.238	.**005**	0.091 (0.025, 0.157)	0.226	.**007**
	Diurnal mean glucose (mg/dL)	0.092 (0.027, 0.156)	0.235	.**005**	0.087 (0.023, 0.152)	0.224	.**008**
	Nocturnal mean glucose (mg/dL)	0.121 (0.041, 0.201)	0.250	.**003**	0.113 (0.033, 0.192)	0.233	.**006**
	Cardiometabolic risk score	10.250 (5.774, 14.726)	0.330	**<**.**001**	10.065 (5.872, 14.257)	0.324	**<**.**001**

The ratio of abdominal IMAT to total abdominal volume was calculated by dividing the abdominal IMAT volume (cm^3^) by the total abdominal cavity volume (cm^3^), thereby accounting for abdominal size. Mid-thigh IMAT is calculated as the sum of IMAT (ie, adipose tissue between the mid-thigh muscles) and IMAT (ie, adipose tissue within the mid-thigh muscles). The ratio of mid-thigh IMAT to total mid-thigh area was calculated by dividing the IMAT area (cm^2^) by the total mid-thigh area (cm^2^), thereby accounting for mid-thigh size. Model 1 represents linear regression analysis adjusted for age and sex, except for the cardiometabolic risk score, which was adjusted only for age. Model 2 represents linear regression analyses adjusted for age, sex, and total fat mass (measured by dual-energy X-ray absorptiometry), except for the cardiometabolic risk score, which was adjusted only for age and total fat mass.

Abbreviations: b, β unstandardized coefficients. β, β standardized coefficients; CI, confidence interval; HOMA-IR, homeostasis model assessment of insulin resistance; IMAT, intermuscular adipose tissue.

Sensitivity analyses were conducted including energy intake and MVPA as covariates in the CRS models [*P* ≤ .001, Supplementary Table S1 (26)], and excluding those participants with impaired fasting glucose [Supplementary Table S2 (26)], and the results remained unchanged [all *P* > .05, Supplementary Table S2 (26)].

The impact of abdominal IMAT/total volume and mid-thigh IMAT/total area on individual cardiometabolic risk factors was analyzed using SEM [Supplementary Figs. S3 and S4 (26)]. The results showed that abdominal IMAT was not significantly associated with the CRS or any of its components. In contrast [all *P* > .05, Supplementary Fig. S3 (26)]. Conversely, mid-thigh IMAT was negatively associated with HDL-C (*P* < .001), fasting glucose (*P* < .001), WC (*P* < .001), SBP (*P* < .001), and DBP (*P* = .006) [Supplementary Fig. S4 (26)].

**Figure 2.**
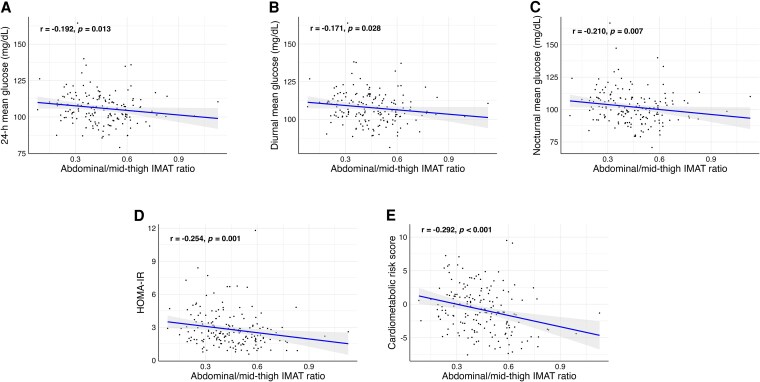
Associations between abdominal/mid-thigh IMAT with 24-hour (A), diurnal (B), and nocturnal mean glucose levels (C); HOMA-IR (D); and cardiometabolic risk score (E). Shading indicates the 95% confidence intervals of the associations. Bold correlations indicate statistically significant associations (*P* < .05). Abbreviations: HOMA-IR, homeostasis model assessment of insulin resistance; IMAT, intermuscular adipose tissue.

**Figure 3.**
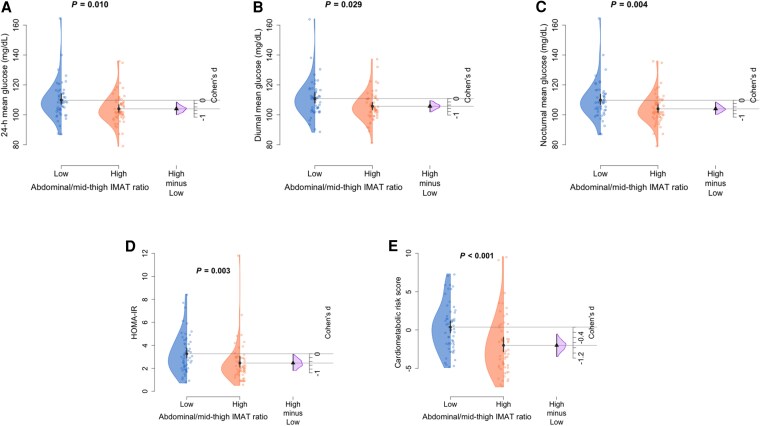
Impact of low vs high abdominal-to-mid-thigh IMAT ratio on 24-hour (A), diurnal (B), and nocturnal mean glucose levels (C); HOMA-IR (D); and cardiometabolic risk score (E). Data are displayed by Gardner–Altman plots, which show a representation of observed values by the 2-group comparison (low abdominal/mid-thigh IMAT ratio and high abdominal/mid-thigh IMAT ratio), a bootstrap effect size (Cohen's d) estimation, mean, and 95% confidence interval. Two-sided *P*-values from a parametric (holistic) or nonparametric (LOWESS) analysis of covariance evaluating the differences between groups while adjusting for sex, except for the cardiometabolic risk score, for which we performed a Krustal–Wallis analysis of variance. Abbreviations: HOMA-IR, homeostasis model assessment of insulin resistance; IMAT, intermuscular adipose tissue.

Abdominal/mid-thigh IMAT ratio was inversely associated with HOMA-IR (*P* = .001, Fig. 2D), 24-hour (*P* = .014, Fig. 2A), and diurnal (*P* = .029, Fig. 2B) and nocturnal mean glucose levels (*P* = .007, Fig. 2C), as well as with CRS (*P* < .001, Fig. 2E). To explore the interplay between these specific fat depots, participants were classified into low and high abdominal/mid-thigh IMAT ratio groups, enabling a more comprehensive assessment of their combined effects on cardiometabolic health (Fig. 3). Participants with a high abdominal/mid-thigh IMAT ratio exhibited lower levels of HOMA-IR (Cohen's d = 0.484, *P* = .003), 24 hour mean (Cohen's d = 0.511, *P* = .010), diurnal (Cohen's d = 0.466, *P* = .029) and nocturnal mean glucose levels (Cohen's d = 0.583, *P* = .004), and CRS (Cohen's d = 0.707, *P* < .001) compared to those with a low abdominal/mid-thigh IMAT ratio.

## Discussion

The results of the present study indicate that mid-thigh IMAT is a stronger contributor to disrupted glucose homeostasis and cardiometabolic risk than abdominal IMAT in adults with overweight or obesity. These findings suggest that specific fat distribution, specifically in the mid-thigh, plays a crucial role in cardiometabolic health, underscoring the importance of targeting specific AT depots in the management and prevention of obesity-related complications. By identifying mid-thigh IMAT as a significant factor, our study contributes to the growing body of evidence that calls for a more nuanced approach to assessing cardiometabolic risk beyond traditional measures.

Several studies indicated that upon adjusting statistical models for BMI or total adiposity, whole-body and mid-thigh IMAT are robust predictors of glycemic control and insulin sensitivity (16, 20). In contrast, the majority of associations between whole-body, abdominal, or thigh IMAT with markers of dyslipidemia disappear when total adiposity is considered (14, 17, 19). Yet, the clinical and physiopathological impact of IMAT may vary depending on the anatomical region, as its effects can be muscle- or muscle compartment-specific, leading to distinct metabolic consequences (30, 31). The results of the present study indicate that, while abdominal IMAT is not associated with insulin resistance and cardiometabolic risk, IMAT accumulation in the mid-thigh region is associated with impaired glycemic homeostasis and cardiometabolic risk in adults with overweight or obesity. Our findings align with previous studies that reported significant associations between mid-thigh IMAT and cardiometabolic risk factors in adults with overweight or obesity but without disruptions in glucose homeostasis (17, 18, 32).

Additionally, the present findings reinforce the notion that a stronger relationship exists between participants with higher IMAT in the lower-body (relative to IMAT in central region) and cardiometabolic risk factors, compared to those with higher IMAT in the central region of the body relative to lower-body IMAT. This is particularly relevant because central obesity and the accumulation of abdominal fat have traditionally been linked to increased cardiometabolic risk (33), contributing to metabolic complications related to obesity (16). However, the specific influence of IMAT located in the abdominal region has been less extensively studied, resulting in inconsistencies in the literature due to variations in individual's characteristics (34). In this context, a higher volume of abdominal IMAT has been linked to an increased prevalence of prediabetes and type 2 diabetes (16, 30).

The results of the present study have important clinical implications, demonstrating that mid-thigh IMAT may serve as a potential imaging marker for the diagnosis and prognosis of cardiometabolic health in adults with overweight or obesity. The protective role of the lower-body fat stores has been confirmed in numerous studies involving subjects with a wide range of ages, BMIs, and comorbidities (35). Gluteo-femoral AT measured by DXA has shown an inverse association with glucose metabolism parameters, including glycated hemoglobin and fasting glucose, as well as with lipid profile (17, 36, 37). However, SAT and IMAT—indistinguishable via DXA—may exhibit independent and even opposing associations with cardiovascular disease risk factors (17). Our findings support this observation, as mid-thigh IMAT shows a significant negative association with mid-thigh SAT [see Supplementary Fig. S1 (26)]. Importantly, these findings highlight the significant role of lower-body IMAT not only in glucose homeostasis but also in other cardiometabolic risk factors. While it seems that IMAT predicts dysglycemia and insulin resistance, its direct and independent effects on other metabolic risk factors remain less extensively studied (3). Additionally, if this important role is confirmed, future research should explore whether lifestyle interventions—particularly exercise training—can effectively promote IMAT reduction and improve cardiometabolic health. Thus, long-term exercise has been shown to nearly double IMAT reduction compared to other interventions, possibly by mobilizing IMAT as a local lipid source for active muscle (3).

The MRI Dixon method employed in this research is recognized as the noninvasive gold standard for quantifying AT, offering excellent image resolution, absence of radiation, high repeatability, and safety (32). Interestingly, several noninvasive imaging techniques, such as muscle ultrasound, have recently emerged as cost-effective and potentially more accessible methods for evaluating muscle quality compared to other imaging techniques (38). However, further exploration is warranted to determine the practical utility of ultrasound techniques in both research and clinical settings (39). Additionally, the significant role of thigh IMAT in cardiovascular health may offer a diagnostic advantage, as assessment of IMAT in the thigh region is more feasible than abdominal evaluation (31). Another important gap in current knowledge relates to the effects of glucagon-like peptide-1 receptor receptor agonists on IMAT. These agents are a novel and effective treatment for weight loss, but the specific changes in body composition associated with their use remain incompletely understood. Some pioneering studies in patients treated with semaglutide (40) have shown that individuals who experienced weight loss exhibited reductions in VAT, SAT, muscle area, and liver fat but did not demonstrate a significant effect on abdominal IMAT. Furthermore, a substantial proportion of patients tend to regain weight over time, often due to treatment discontinuation within the first year (41). In such cases, those who gained weight after treatment showed increases in IMAT, along with decreased muscle mass.

Given the emerging role of mid-thigh IMAT as a marker of metabolic dysfunction and physical decline, it is crucial to further investigate this depot, particularly through strategies specifically targeting mid-thigh IMAT and elucidating its impact on cardiometabolic risk.

Finally, some limitations should be acknowledged. First, as this is a cross-sectional study, causality cannot be established. Second, the study population consists of participants of Caucasian descent, limiting the generalizability of the results to other racial or ethnic groups. Racial differences in the distribution of IMAT may contribute to variations in dysglycemia (9). Future multicenter studies are needed to establish significant correlations between IMAT depots and cardiovascular risk factors across diverse populations. In the present study, 2 CGM device models were used, acknowledging that accuracy may vary between models. Anyway, current devices show improved performance—often comparable to blood glucose monitoring systems—accuracy still depends on glucose levels and their rate of change (42). Our analysis focused on nondiabetic individuals with relatively stable glucose levels, a context in which FreeStyle Libre devices have demonstrated accuracy comparable to other real-time CGM systems (43).

In conclusion, the findings of this study suggest that the relationship between IMAT distribution, glucose homeostasis, and cardiometabolic risk varies by anatomical location. Specifically, higher IMAT in the lower body may serve as a more reliable predictor of cardiometabolic risk than abdominal IMAT in adults with overweight or obesity. Future studies including participants across a broad range of ages and comorbidities are required, particularly those with impaired glucose homeostasis.

## Data Availability

Due to privacy concerns, the datasets used in this study are not publicly available; however, researchers can request access to specific individual-level data for academic use only. Proposals should be directed to corresponding authors J.R.R. and A.C.C. Upon proposal acceptance, requesters will be granted access to the data after signing a data access agreement.

## Clinical Trial Information

NCT05310721.
