# Correction to: “Impact of Abdominal and Thigh Intermuscular Adipose Tissue on Glucose and Cardiometabolic Risk in Adults With Obesity”

**DOI:** 10.1210/clinem/dgaf600

**Published:** 2025-11-14

In the above-named article by Camacho-Cardenosa A, Clavero-Jimeno A, Gatti A, Dote-Montero M, Concepción M, Alfaro-Magallanes VM, Martin-Olmedo JJ, Cabeza R, Idoate F, Martín-Rodríguez JL, García Pérez PV, Muñoz-Torres M, Ruiz JR, and Labayen I (*J Clin Endocrinol Metab*. 2025; doi: 10.1210/clinem/dgaf362) there was an error in the Funding section.

In the originally published article, in the Funding section, the first sentence read as, “Agencia Estatal de Investigación, Ministerio de Ciencia, Innovación y Universidades grant PID2022.141506OB.I00 (J.R.R.).” In the corrected article, the sentence now reads, “Grant PID2022-141506OB-I00 funded by MICIU/AEI /10.13039/501100011033 and by ERDF, EU. (JRR).”

The article has been corrected online.

doi: 10.1210/clinem/dgaf362

